# Heritability of the dimensions, compliance and distensibility of the human internal jugular vein wall

**DOI:** 10.1371/journal.pone.0192948

**Published:** 2018-03-21

**Authors:** Adam Domonkos Tarnoki, Andrea Agnes Molnar, David Laszlo Tarnoki, Levente Littvay, Emanuela Medda, Corrado Fagnani, Antonio Arnolfi, Filippo Farina, Claudio Baracchini, Giorgio Meneghetti, Giacomo Pucci, Giuseppe Schillaci, Maria Antonietta Stazi, György L. Nadasy

**Affiliations:** 1 Department of Radiology, Semmelweis University, Budapest, Hungary; 2 Vascular Heart Center, Semmelweis University, Budapest, Hungary; 3 Central European University, Budapest, Hungary; 4 Genetic Epidemiology Unit, National Centre of Epidemiology, Istituto Superiore di Sanità, Viale Regina Elena, Rome, Italy; 5 Department of Neurosciences, University of Padua School of Medicine, Padova, Italy; 6 Università di Perugia, Unità di Medicina Interna, Ospedale "S. Maria", Terni, Italy; 7 Department of Physiology, Semmelweis University, Budapest, Hungary; University of Modena and Reggio Emilia, ITALY

## Abstract

**Aims:**

The elasticity of the internal jugular vein (IJV) is a major determinant of cerebral venous drainage and right atrium venous return. However, the level of genetic determination of IJV dimensions, compliance and distensibility has not been studied yet.

**Methods:**

170 adult Caucasian twins (43 monozygotic [MZ] and 42 dizygotic [DZ] pairs) were involved from the Italian twin registry. Anteroposterior and mediolateral diameters of the IJV were measured bilaterally by ultrasonography. Measurements were made both in the sitting and supine positions, with or without Valsalva maneuver. Univariate quantitative genetic modeling was performed.

**Results:**

Genetic factors are responsible for 30–70% of the measured properties of IJV at higher venous pressure even after adjustment for age and gender. The highest level of inheritance was found in the supine position regarding compliance (62%) and venous diameter during Valsalva (69%). Environmental and measurement-related factors instead are more important in the sitting position, when the venous pressure is low and the venous lumen is almost collapsed. The range of capacity changes between the lowest and highest intraluminal venous pressure (full distension range) are mainly determined by genetic factors (58%).

**Conclusions:**

Our study has shown substantial heritability of IJV biomechanics at higher venous pressures even after adjustment for age and gender. These findings yield an important insight to what degree the geometric and elastic properties of the vascular wall are formed by genetic and by environmental factors in humans.

## Introduction

Large veins have a greater distensibility and compliance within the physiological pressure range compared to arteries, which helps keep venous pressure within the normal range [1–2]. The biomechanical properties of veins depend on age, gender, and body mass; gravitation is also a determinant as these features change according to the localization of veins and body position [3–6]. Pathologic hemodynamic factors together with local and generalized inflammatory processes might lead to venous wall remodeling and alterations of venous biomechanics, which further contribute to venous hypertension and progression of venous dysfunction [7–8]. There is a limited number of studies on the biomechanical properties of human veins in vivo, and few earlier publications investigated the elasticity of IJV [6, 9–12]. This vein has special elastic properties, different from the more frequently studied lower extremity veins, due to its opposite localization with respect to the right atrium [6]. In the supine position, the intraluminal pressure of IJV steeply increases and the distensibility of the venous wall decreases [6], forming most of the cerebral venous drainage. Indeed, this occurs in the majority of normal subjects (about 70%), called jugular drainers. In the remaining 30% of the normal population draining occurs via the vertebral veins, deep neck veins or via the intraspinal venous system[13]. Changing to a sitting or upright position, IJV distensibility increases as the transmural pressure approaches 0 mmHg; as a consequence, IJV collapses and blood flow resistance increases. In these positions, a larger part of the cerebral venous drainage is ensured by the vertebral venous system [14].

Undoubtedly, the elasticity of IJV is a major determinant of cerebral venous drainage and right atrium venous return. An impaired IJV elasticity is thought to determine a reduction of cerebral venous outflow, retrograde venous hypertension, and venous congestion of the brain leading to several neurological disorders such as transient global amnesia and chronic cerebrospinal venous insufficiency [2]. Other authors have questioned these links [15–18] explaining that an increased IJV wall stiffness leads to a diversion of flow towards the vertebral venous plexus thus avoiding an increase of intracranial pressure according to the Starling resistor model of venous circulation.

In an earlier study on monozygotic and dizygotic twins, we separated the inherited, shared environmental and unshared environmental factors in forming the geometric and elastic properties of the common femoral vein [19]. However, the level of genetic determination of IJV biomechanics has not been studied yet. The aim of our present work was to evaluate the heritability of geometric and elastic properties of IJV in a population of healthy twins. Such observations can shed more light on the venous side of the cerebral circulation, a not well studied area, as well as may yield new data concerning the role of inheritance of the elastic properties of veins in general.

## Materials and methods

### Subjects and study design

170 adult Caucasian twins (43 monozygotic [MZ] and 42 dizygotic [DZ] pairs) included in the Italian twin registry were invited to participate in this cross-sectional multicentric twin study [20] in Padua, Perugia and Terni. All such twins were contacted and invited by mail by the members of the Italian twin registry and the investigations were described in detail with the exclusion criteria. The recruitment was designed to balance the overall participation for 50% females and at least 50% DZ twins. Exclusion criteria included history of serious cardiovascular and respiratory disease, pregnancy, acute infection within three weeks and foreseeable lack of compliance with test procedures. Chronic cardiovascular or pulmonary disease was excluded using questionnaire, personal interview and echocardiography. Study subjects were requested to take their medical documents. If these charts were provided, they were reviewed on the spot. All subjects were asked not to smoke three hours, not to eat one hour, not to drink alcohol and coffee ten hours prior to the measurements in order to exclude the effects of these factors on the vascular tone. A multiple self-reported questionnaire was used to maximize the accuracy of zygosity classification [21]. The protocol of this observational study was under the general framework of the Italian Twin Registry research activities, approved by the Ethics Committee of the Istituto Superiore di Sanità. All study subjects gave informed consent prior to entering the study after explanation of the nature and possible consequences of the study, which was conducted in full compliance with regulations of local ethical committees. The tenets of the Declaration of Helsinki were followed.

The vascular measurements were obtained at three large hospitals in Italy: Padua, Perugia and Terni. Participants were asked to fill in an additional questionnaire in order to report complete past medical history, risk factors and current morbidities. Weight measurements were carried out by a clinically validated OMRON BF500 body consistency monitor (Omron Healthcare Ltd., Kyoto, Japan). Current height was verified simultaneously in order to calculate the body mass index (BMI).

### Jugular ultrasonography

Anteroposterior and mediolateral diameters of IJV were measured bilaterally by high-resolution color-coded duplex sonography scanners (in Padua: Toshiba Aplio XG, in Perugia: Sonoscape S8, in Terni: Esaote MyLab 60) using high-frequency (5–10 MHz) linear probes. The image resolution (0.1 mm) provided by the high frequency probes was sufficient to track diameter changes with sufficient accuracy. The examination was performed in a quiet atmosphere, with the subjects lying first in a sitting position and then in a supine position. Each subject was asked to place his head in a straight position in order to avoid flow alterations caused by unilateral or bilateral venous outflow obstruction. Great care was taken not to compress the IJV when the probe was applied over the neck, in order to obtain reliable diameter measurements. IJVs’ diameters were measured at the level of the middle portion of the common carotid artery, approximately 5–7 cm above the junction with the subclavian vein. Measurements were made first at rest and then during a Valsalva maneuver: the patients were asked to produce a forced expiration while holding a tube in their mouth connected to an electromanometer until the requested pressure (60 mmHg) was reached and maintained for a few seconds. All IJV images were saved and for each subject both anteroposterior and mediolateral diameters were measured by built-in calipers. Those subjects (ladies, elderly twins) who could not reach the requested pressure values (60 mmHg) were excluded. The original measured data are shown in Supplementary S1 Table. The recorded data was used to calculate capacity per unit length according to the formula d_1_*d_2_*3.142/4, in which d_1_ is the anteroposterior, d_2_ is the mediolateral diameter. Full distension range was calculated by the difference of capacities taken at the highest (supine 60 mmHg) and lowest (sitting 0 mmHg) pressures. Venous compliance was defined a change in blood volume (ΔV) within a vein associated with a change in intravenous distending pressure (ΔP). Venous distensibility was defined as the percent increase in venous volume per mmHg increase in pressure.

### Statistical analysis

A descriptive analysis (mean standard deviation, and percentage for categorical variables) for risk factors and jugular parameters was conducted by SPSS Statistics 17. P values less than 0.05 were considered significant by the comparison of two groups with independent samples t-test.

Structural equation modeling was performed by using the Mplus Version 7.1 (Muthén & Muthén, Los Angeles, CA, USA) maximum likelihood estimation [22]. The classical twin study investigates both MZ and DZ twin pairs since the consideration is that greater levels of MZ than DZ within pair correlation indicate a genetic influence on a phenotype, while similarity of co-twin correlations suggests that the variance is due to shared environmental sources. Larger similarity between DZ than MZ twins indicates that the variance is due to the unshared environmental components. We know that identical twins share their genome (r = 1) while this correlates r = 0.5 for fraternal twins. We also know that on average, both MZ and DZ twins equally share their common environment (r = 1 for both MZ and DZ twins). The unique environment of the co-twins remains uncorrelated for both zygosities. Accordingly, univariate quantitative genetic modeling was performed to decompose the phenotypic variance of the considered parameters into heritability (A), shared (C), and unshared (E) environmental effects (ACE analysis) [23]. In the structural equation model A, C and E components are latent variables but for both co-twins these latent variables are related to each other based on the described structure giving us the ability to estimate the proportions of interest. The additive genetic component (A) measures the effect due to genes at multiple loci or multiple alleles at one locus. The shared environmental component (C) estimates contribution of a common family environment for both twins (e.g., familiar socialization, shared womb), whereas the unshared environmental component (E) estimates the effects that separately apply to each individual twin and accounts for measurement errors. The ACE model has been applied with age and sex adjustments regressed out of the phenotype simultaneously with the ACE structural equation model estimation. Adjustments for height, BMI and smoking have been tested but no correlation with the measured venous parameters has been observed. Data without such adjustments will be shown in the Results. In order to find the most parsimonious model for the investigated jugular traits, besides the fully-fledged ACE models, sub-models were also constructed. The AE model discards the common environmental effects (C), the CE sub-model disregards the additive genetic effects (A), while the E model summarizes all effects, which are completely uncorrelated between twin pairs. Homogeneity of the full ACE and the sub-models were assessed with χ^2^ tests. If one of the reduced models fit significantly worse based on the likelihood ratio chi-sqaure test, we accepted the model that was not significantly worse than the full ACE. If neither of the AE or CE models fit worse, we accepted the ACE model. Empirical 95% confidence intervals were calculated with a Bollen-Stine Bootstrap [24]. All inferential statistics were estimated using full information maximum likelihood.

## Results

### Subject characteristics

Baseline characteristics of all study subjects and the subgroup of monozygotic and dizygotic twins according to zygosity are presented in *Table 1.* The majority (57.5%) of study subjects were women. Mean age was 45.1±13.6 years. Prevalence of hypertension and smoking was 14.3% and 42.9%, respectively, while mean BMI was 24.8±4.2 kg/m^2^. No significant difference in these parameters was observed between MZ and DZ twins.

**Table 1:** Baseline characteristics of study subjects.

	Total (n = 170)	Monozygotic (n = 86)	Dizygotic (n = 84)	*P* value
Male:female	71:99	41:45	30:54	0.115
Age, years	45.1±13.6	44.5±12.9	45.8±14.6	0.558
BMI, kg/m^2^	24.8±4.2	24.3±3.8	25.2±4.6	0.143
Hypertension, n (%)	24 (14.3)	10 (12.0)	14 (16.9)	0.380
Diabetes, n (%)	2 (1.2)	0 (0.0)	2 (2.5)	0.159
Hypercholesterolemia, n (%)	22 (13.5)	13 (15.5)	9 (10.7)	0.363
Smoking, n (%)	71 (42.9)	32 (37.6)	39 (47.0)	0.223
Full distension range, left	140.8±80.6	139.1±87.8	141.3±72.7	0.865
Full distension range, right	238.8±123.3	250.0±136.8	224.7±107.4	0.181
Full distension range, average	191.6±87.1	195.7±97.1	185.5±75.7	0.451

BMI, body mass index. Data are shown as mean ± standard deviation where appropriate.

### Heritability analysis

**Fig 1:**
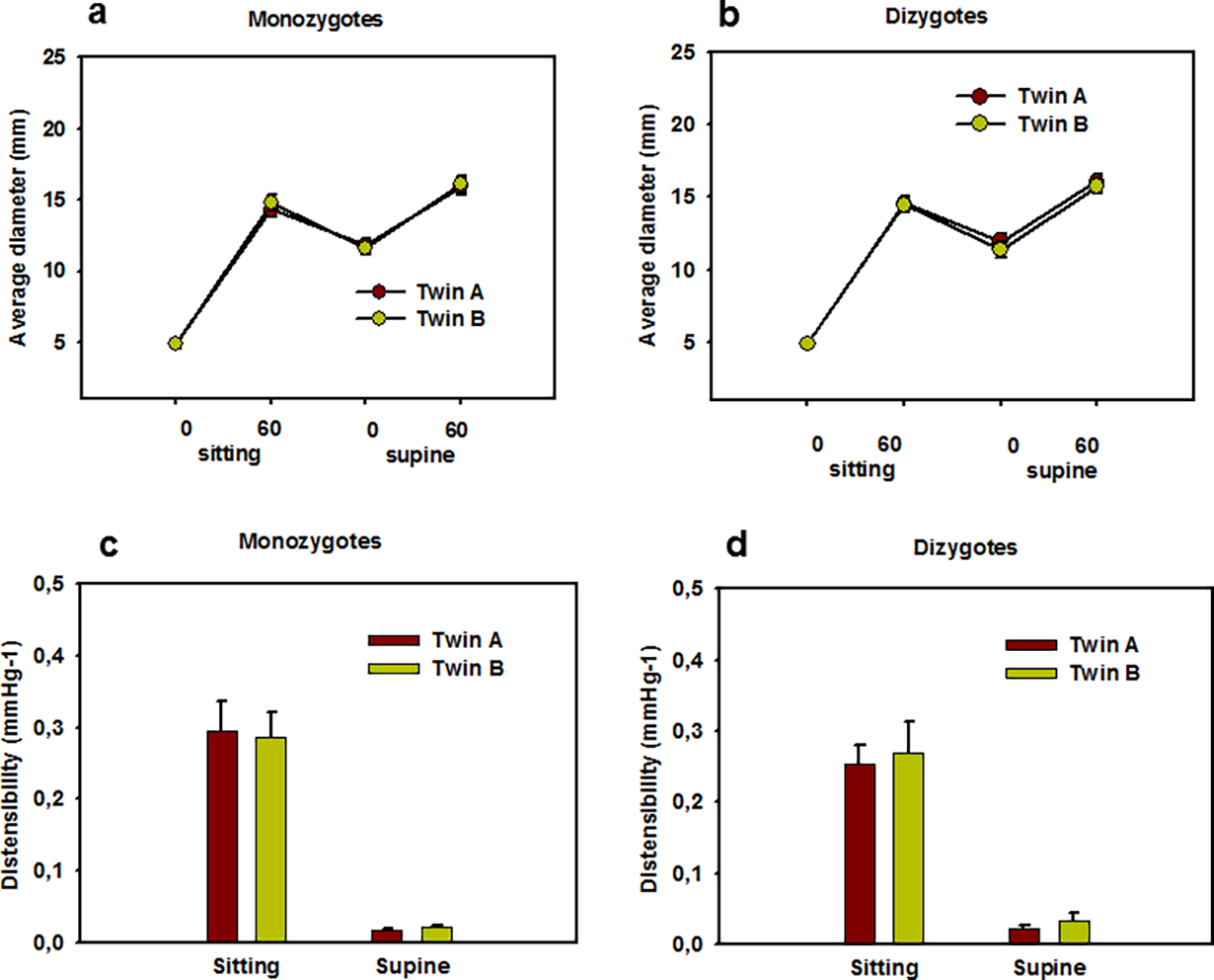
Inner jugular vein diameters measured ultrasonographically in monozygotic (a) and dizygotic (b) A and B twins, in the sitting and in the supine body position, without and with a controlled 60 mmHg Valsalva. Antero-posterior and lateral diameters were averaged for the left and right sides for each person. Note very close mean values for two members of the twin pairs in both groups. Distensibilities computed for monozygotic (c) and dizygotic (d) A and B twins. Distensibilities for the left and right inner jugular veins measured between 0–60 mmHg Valsalva were averaged for each person.

**Fig 2:**
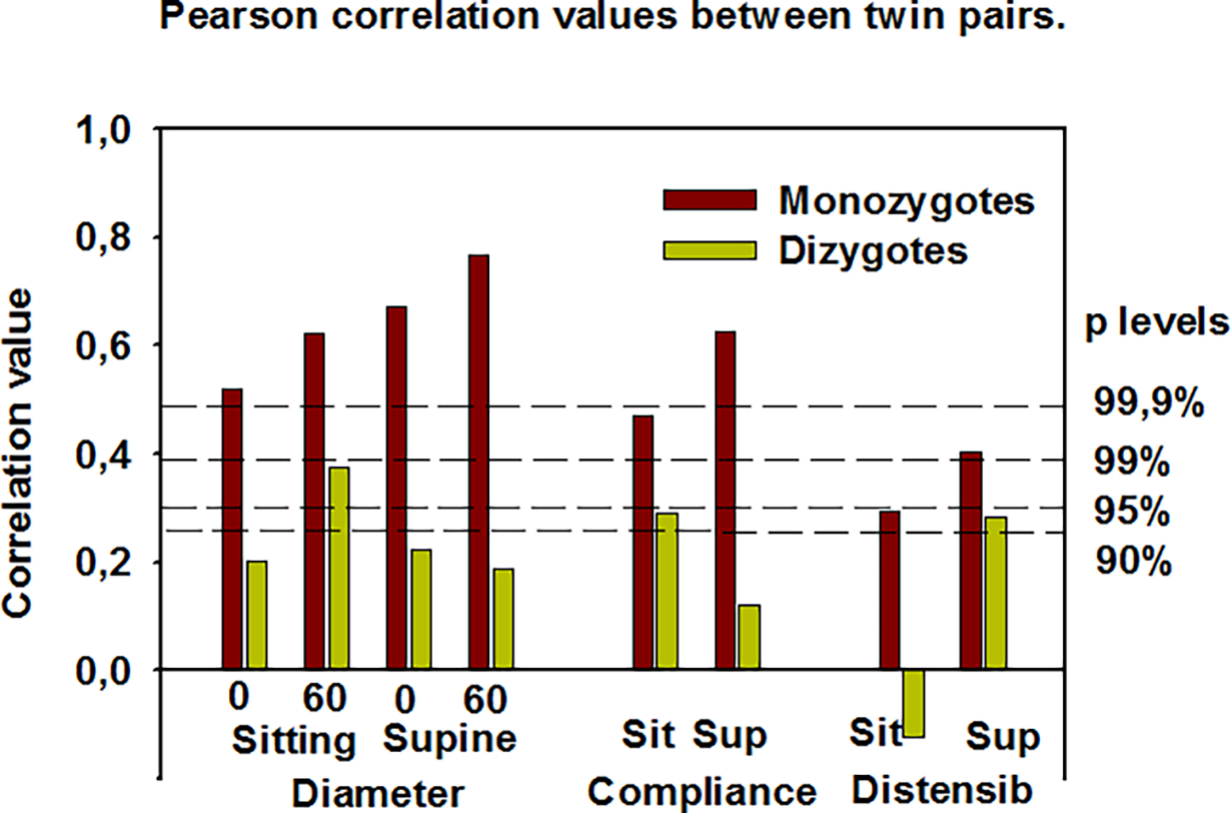
Intertwin correlation of average diameter, compliance and distensibility for monozygotic and ditzygotic twins in the sitting and in the supine positions without and with a 60 mmHg controlled Valsalva attempt. At right the significance levels of the correlation coefficients are shown (valid for monozygotes, n = 40, but almost identical for dizygotes, n = 42). Note higher correlation values for monozygotic twins (hinting for genetic factors). Note limited correlation of values sitting (measured at lower initial pressure).

Geometrical and elasticity values of the IJV were very similar for twin pairs both in MZ and DZ twins (Fig 1). However, co-twin correlations were regularly higher for MZ than for DZ twin pairs (Fig 2). The original measured anonymized data can be found in Supplementary S1 Table.

Structural equation modeling was used to further decompose the variance between the twins to additive genetic, shared and unshared environmental components. Basic model was constructed with adjustment for sex and age. Heritability estimates (see values ‘A’ in Table 2) for jugular vein diameter in sitting position without Valsalva was low (30%) but statistically significant. In this state, when the gravitation empties the jugular vein, our model marks a substantial unshared environmental component (‘E’). In the supine position, when intraluminal pressure increases, the genetic component in the variance of the diameter is a remarkable 56% (with 95% confidence intervals between 35–72%), and even higher (69%) during Valsalva (rigid, maximally dilated diameter).

**Table 2:** Age and sex adjusted parameter estimates for additive hereditary (A), common environment (C) and unique environmental influences (E) of mean internal jugular vein diameter by structural equation modeling.

	Models	A	95% CI	C	95% CI	E	95% CI
Average of the two sides							
**Sitting** **0 Hgmm Model 1**	A-C-E*	**0.304**	0.005–0.586	0.000**	0.000–0.333	**0.696**	0.418–0.945
	A-E	**0.304**	0.051–0.582	**0.000**	0.00–0.00	**0.696**	0.417–0.949
	C-E	**0.000**	0.000–0.000	0.197**	0.000–0.443	**0.803**	0.550–1.000
**Sitting** **60 Hgmm Model 1**	A-C-E*	0.355**	0.000–0.715	0.238**	0.000–0.657	**0.406**	0.215–0.639
	A-E	**0.605**	0.376–0.789	**0.000**	0.000–0.000	**0.395**	0.209–0.622
	C-E	**0.000**	0.000–0.000	**0.523**	0.322–0.700	**0.477**	0.299–0.676
**Supine** **0 Hgmm Model 1**	A-C-E	**0.561**	0.352–0.737	**0.000**	0.000–0.138	**0.439**	0.272–0.647
	A-E*	**0.561**	0.352–0.724	**0.000**	0.000–0.000	**0.439**	0.273–0.647
	C-E	**0.000**	0.000–0.000	**0.353**	0.194–0.537	**0.647**	0.458–0.805
**Supine** **60 Hgmm Model 1**	A-C-E	**0.693**	0.370–0.852	**0.000**	0.000–0.568	**0.307**	0.178–0.481
	A-E*	**0.693**	0.524–0.822	**0.000**	0.000–0.000	**0.307**	0.178–0.476
	C-E	**0.000**	0.000–0.000	**0.550**	0.393–0.691	**0.450**	0.308–0.607

*most parsimonious, best fitting model

**insignificant values

CI: confidence interval. MZ, monozygotic; DZ, dizygotic; A, heritability; C, shared environmental variance component

E, unique environmental variance component.

Similar observations were made for venous compliance (Table 3). Volume elevation in the supine position in response to a 60 mmHg Valsalva maneuver has shown a significant inherited component (‘A’, 61.5%, with confidence intervals of 37–79%) but the heritability was insignificant in sitting position. Inheritance in the distensibility (same supine position) was 41%, demonstrating that the ability for relative volume elevation in response to pressure is at least partially genetically determined. A-E was the most parsimonious, best fitting model indicating a 58% heritability for full distension range, the parameter that marks the distance between the elastically least stressed and most stressed diameters.

**Table 3:** Age and sex adjusted parameter estimates for additive hereditary (A), common environment (C) and unique environmental influences (E) of compliance, distensibility and full distension range (values averaged for both sides) by structural equation modeling.

	Models	A	95% CI	C	95% CI	E	95% CI
**Sitting compliance**	A-C-E*	0.018**	0.000–0.661	0.404**	0.000–0.704	**0.578**	0.344–0.881
	A-E	**0.448**	0.166–0.711	**0**	0.000–0.000	**0.552**	0.287–0.832
	C-E	**0**	0.000–0.000	**0.419**	0.184–0.661	**0.581**	0.337–0.816
**Supine compliance**	A-C-E	**0.615**	0.302–0.808	0.000**	0.000–0.401	**0.385**	0.212–0.628
	A-E*	**0.615**	0.372–0.788	**0**	0.000–0.000	**0.385**	0.211–0.627
	C-E	**0**	0.000–0.000	**0.46**	0.221–0.653	**0.54**	0.347–0.778
**Distensibility**	A-C-E	**0.41**	0.101–0.683	**0**	0.000–0.000	**0.59**	0.317–0.897
	A-E*	**0.41**	0.095–0.673	**0**	0.000–0.000	**0.59**	0.318–0.904
	C-E	**0**	0.000–0.000	0.17**	0.000–0.412	**0.826**	0.588–1.000
**Full distension range**	A-C-E	**0.581**	0.008–0.849	0.000**	0.000–0.417	**0.419**	0.181–0.653
	A-E*	**0.581**	0.341–0.817	**0**	0.000–0.000	**0.419**	0.182–0.659
	C-E	**0**	0.000–0.000	**0.438**	0.233–0.650	**0.562**	0.350–0.766

*most parsimonious, best fitting model

**insignificant values

CI: confidence interval. MZ, monozygotic; DZ, dizygotic; A, heritability; C, shared environmental variance component

E, unique environmental variance component

## Discussion

The aim of the present study was to determine the heritability of static and dynamic biomechanical properties of IJV in a cohort of adult Caucasian twin population. Non-invasive ultrasonographic technique was used combined with controlled Valsalva maneuver and postural change to determine venous dimensions, compliance and distensibility. To our knowledge, no study was previously performed to estimate heritability of IJV geometry and elasticity. Classical twin studies are extensively used to characterize the interaction of genetic and environmental factors on determining vascular phenotypes [25]. Heritability reflects how much the variation of the properties of IJV is due to variation in genetic factors.

The main finding of our work is that genetic factors are responsible for 30–70% in determining the geometric and elastic properties of IJV at higher venous pressure even after adjustment for age and sex. The highest level of age, sex and smoking adjusted inheritance was found in the supine position regarding compliance (62%) and venous diameter during Valsalva pressure (69%). Environmental and measurement-related factors instead are more important in the sitting position, when the venous pressure is low and the venous lumen is almost collapsed. The range of capacity changes between the lowest and highest intraluminal venous pressure (full distension range) also proved to be determined mainly by genetic factors (58%). This represents the heritability of IJV compliance between the sitting position at rest (without Valsalva maneuver) and the supine position during Valsalva maneuver.

Few earlier publications reported on biomechanical behavior of IJV [6,9–12]. In the supine position, the IJV dilates as its intraluminal pressure increases. Further dilatation can be reached by a Valsalva maneuver. Conversely, the IJV diameter in the sitting or erect position decreases significantly as blood gushes down from it into the right atrium, reducing transmural pressure to zero or below. Thus additional factors, such as the course of the vein and the surrounding tissue may affect the biomechanical behavior of IJV, each bearing its own inheritance. Further determining factors of in situ venous mechanics include local myogenic and humoral mechanisms of venous wall as well as systematic humoral and nervous control on veins [5–6]. Several pathological conditions, like chronic heart failure, pulmonary hypertension and chronic obstructive pulmonary disease can elevate central venous pressure, but no such disease was present in our subjects [26].

The role of extracranial venous system in different pathological processes is largely unknown. The IJVs are the largest veins in the neck of humans and generally considered the most important cerebral venous outflow pathway [2]. The significance of venous cerebral structural and functional variations and abnormalities are still lacking. Distensibility of IJV helps keep venous pressure within normal values. In case of reduced distensibility, the IJV loses its compensatory ability to increased transmural pressure and it becomes prone to venous hypertension. Chung and coworkers reported on decreased IJV distensibility in subjects suffering of migraine [27]. Triggering factors of migraine would increase sympathetic tone which could increase the venous tone and pressure which might be transmitted to cerebral venous circulation leading to headache attack. The role of venous biomechanics in aeronautics is another active research area, including the potential of vision impairment hypothesized to be caused by increased intracranial pressure secondary to the headward fluid shift caused by microgravity [28–29].

Our study has to be interpreted within the context of its potential limitations. The effect of gene-to-gene and gene-to-environment interplay was not included in the model; however, it can modify the heritability estimates. Other basic assumption of the classic twin model is that MZ and DZ environments are the same, but this is not always the case. Further limitations include the relatively small number of participating dizygotic twins compared to usual twin studies, which may lead to statistical errors in the ACE analysis by increasing the E variance. It is important to note that our estimates of heritability are not an absolute measurement of how genes and environment determine properties of IJV, but specific to the twin population and environment of our study. Because our study sample included adult Italian individuals without relevant known diseases only, the extent to which our findings may apply to other age and ethnic groups remains unknown.

Chronic cardiovascular or pulmonary disease was excluded using questionnaire, personal interview and echocardiography, but no spirometry was performed which is an additional limitation of our study. If study subjects did not take their medical documents and the disease was not revealed during the interview or in the questionnaire, some chronic cardiovascular or pulmonary conditions might be missed as exclusion criteria. However, we suspect that the number of such cases was negligible in our cohort.

In summary, our study demonstrated substantial heritability of the dimensions, compliance and distensibility of IJV at higher venous pressures even after adjustment for age and gender. These findings provide support for further research on genes affecting IJV biomechanics at higher venous pressures. Our data provide further data to understand the venous side of the human brain circulation as well as the role of inheritance in the formation of geometric and elastic properties of veins.

## Supporting information

S1 Table Diameters of the inner jugular vein measured ultrasonographically. **Original data measured on twins.** Anonimous Italian twin registry codes are shown. F, female, M, male, MZ, monozygotic, DZ, dizygotic twin pairs. Sit, sitting, Sup, supine body positions. Vals, during 60 mmHg Valsalva test, no Vals, without it with normal breathing. MLD, mediolateral diameter, APO anteroposterior diameter, in mm.
(PDF)

## References

[1] Sheperd JT, Vanhoutte PM. Veins and their control WB. Saunders, Philadelphia, 1975, pp. 1–269.

[2] Zivadinov R, Chung CP. Potential involvement of the extracranial venous system in central nervous system disorders and aging. BMC Medicine, 2013; 11:260 doi: 10.1186/1741-7015-11-260 24344742 10.1186/1741-7015-11-260 PMC3866257

[3] Olsen H, Lanne T. Reduced venous compliance in lower limbs of aging humans and its importance for capacity function. Am J Physiol 1998; 275:H878–H886. 9724292 10.1152/ajpheart.1998.275.3.H878

[4] Fronek A, Criqui MH, Denenberg J, Langer RD. Common femoral vein dimensions and hemodynamics including Valsalva response as a function of sex, age, and ethnicity in a population study. J Vasc Surg 2001; 33:1050–1056. doi: 10.1067/mva.2001.113496 11331848 10.1067/mva.2001.113496

[5] Monos E, Lorant M, Dornyei G, Berczi V, Nadasy G. Long-term adaptation mechanisms in extremity veins supporting orthostatic tolerance. News Physiol Sci 2003; 18:210–214. 14500802 10.1152/nips.01447.2003

[6] Berczi V, Molnar AA, Apor A, Kovacs V, Ruzics C, Varallyay C, Huttl K, Monos E, Nadasy GL. Non-invasive assessment of human large vein diameter, capacity, distensibility and ellipticity in situ: dependence on anatomical location, age, body position and pressure. Eur J Appl Physiol 2005; 95:283–289. doi: 10.1007/s00421-005-0002-y 16151839 10.1007/s00421-005-0002-y

[7] Molnár AA, Apor A, Kristóf V, Nádasy GL, Préda I, Hüttl K, Acsády G, Monos E, Bérczi V. Generalized changes in venous distensibility in post¬thrombotic patients. Thromb Res 2006; 117:639–645. doi: 10.1016/j.thromres.2005.05.012 16019057 10.1016/j.thromres.2005.05.012

[8] Molnár AA, Apor A, Kristóf V, Nádasy GL, Szeberin Z, Monos E, Acsády G, Préda I, Bérczi V. Generalized alterations in the biomechani¬cal properties of large veins in non-thrombotic thrombophilic young patients. Intl Angiol 2008; 27:247–252. 18506128

[9] Armstrong PJ, Sutherland R, Scott DH. The effect of position and different manoeuvres on internal jugular vein diameter size. Acta Anaesthesiol Scand 1994; 38:229–231. 8023661 10.1111/j.1399-6576.1994.tb03879.x

[10] Attubato MJ, Katz ES, Feit F, Bernstein N, Schwartzman D, Kronzon I. Venous changes occurring during the Valsalva maneuver: evaluation by intravascular ultrasound. Am J Cardiol 1994; 74:408–410. 8059711 10.1016/0002-9149(94)90417-0

[11] Lobato EB, Florete OG Jr, Paige GB, Morey TE. Cross-sectional area and intravascular pressure of the right internal jugular vein during anesthesia: effects of Trendelenburg position, positive intrathoracic pressure, and hepatic compression. J Clin Anesth 1998; 10:1–5. 9526929 10.1016/s0952-8180(97)00189-x

[12] Botero M, White SE, Younginer JG, Lobato EB. Effects of Trendelenburg position and positive intrathoracic pressure on internal jugular vein cross-sectional area in anesthetized children. J Clin Anesth 2001; 13:90–93. 11331166 10.1016/s0952-8180(01)00220-3

[13] Doepp F, Schreiber SJ, von Munster T, Rademacher J, Klingebiel R, Valdueza JM. How does the blood leave the brain? A systematic ultrasound analysis of cerebral venous drainage patterns. Neuroradiology 2004; 46:565–570. doi: 10.1007/s00234-004-1213-3 15258709 10.1007/s00234-004-1213-3

[14] Valdueza JM, von Munster T, Hoffman O, Schreiber S, Einhaupl KM. Postural dependency of the cerebral venous outflow. Lancet 2000; 355:200–201. 10.1016/s0140-6736(99)04804-7 10675123

[15] Baracchini C, Perini P, Calabrese M, Causin F, Rinaldi F, Gallo P. No evidence of chronic cerebrospinal venous insufficiency at multiple sclerosis onset. Ann Neurol 2011; 69:90–99. doi: 10.1002/ana.22228 21280079 10.1002/ana.22228

[16] Baracchini C, Perini P, Causin F, Calabrese M, Rinaldi F, Gallo P. Progressive multiple sclerosis is not associated with chronic cerebrospinal venous insufficiency. Neurology 2011; 77:844–850. doi: 10.1212/WNL.0b013e31822c6208 21849656 10.1212/WNL.0b013e31822c6208

[17] Baracchini C, Tonello S, Farina F. Jugular veins in transient global amnesia: innocent bystanders. Stroke 2012; 43:2289–2292. doi: 10.1161/STROKEAHA.112.654087 22811457 10.1161/STROKEAHA.112.654087

[18] Lochner P, Nedelmann M, Kaps M, Stolz E. Jugular valve incompetence in transient global amnesia. A problem revisited. J Neuroimaging 2014; 24:479–83 doi: 10.1111/jon.12042 24033644 10.1111/jon.12042

[19] Molnár AÀ, Tárnoki AD, Tárnoki DL, Kulcsár Z, Littvay L, Garami Z, Préda I, Kiss RG, Bérczi V, Lannert A, Monos E, Nádasy GL. Heritability of venous biomechanics. Arterioscler Thromb Vasc Biol 2013; 33:152–157. doi: 10.1161/ATVBAHA.112.300062 23117659 10.1161/ATVBAHA.112.300062

[20] Brescianini S, Fagnani C, Toccaceli V, Medda E, Nisticò L, D'Ippolito C, Alviti S, Arnofi A, Caffari B, Delfino D, Ferri M, Penna L, Salemi M, Sereni S, Serino L, Cotichini R, Stazi MA. An update on the Italian Twin Register: advances in cohort recruitment, project building and network development. Twin Res Hum Genet 2013; 16:190–196. doi: 10.1017/thg.2012.85 23088847 10.1017/thg.2012.85

[21] Heath AC, Nyholt DR, Neuman R, Madden PA, Bucholz KK, Todd RD, Nelson EC, Montgomery GW, Martin NG. Zygosity diagnosis in the absence of genotypic data: an approach using latent class analysis. Twin Res 2003; 6:22–26. 12626225 10.1375/136905203762687861

[22] Muthén LK, Muthén BO. Mplus. Statistical Analysis with Latent Variables User’s Guide (1998–2010). Los Angeles: Muthén & Muthén 2010; 5:84–85.

[23] Jinks JL, Fulker DW. Comparison of the biometrical genetical, MAVA, and classical approaches to the analysis of human behavior. Psychol Bull 1970; 73:311–349. 5528333 10.1037/h0029135

[24] Bollen KA, Stine RA. Bootstrapping goodness-of-fit measures in structural equation models. Sociol Methods Res 1992; 201:205–229.

[25] Friedrich CL. Twins in cardiovascular genetic research. Hypertension 2001; 37:350–356. 11230299 10.1161/01.hyp.37.2.350

[26] Doepp F, Bähr D, John M, Hoernig S, Valdueza JM, Schreiber SJ. Internal jugular vein valve incompetence in COPD and primary pulmonary hypertension. J Clin Ultrasound 2008; 36:480–484. doi: 10.1002/jcu.20470 18335510 10.1002/jcu.20470

[27] Chung CP, Chao AC, Hsu HY, Lin SJ, Hu HH. Decreased jugular venous distensibility in migraine. Ultrasound Med Biol 2010; 36:11–16. doi: 10.1016/j.ultrasmedbio.2009.08.007 19900748 10.1016/j.ultrasmedbio.2009.08.007

[28] Arbeille P, Fomina G, Roumy J, Alferova I, Tobal N, Herault S. Adaptation of the left heart, cerebral and femoral arteries, and jugular and femoral veins during short- and long-term head-down tilt and space-flights. Eur J Appl Physiol 2001; 86:157–168. doi: 10.1007/s004210100473 11822475 10.1007/s004210100473

[29] Aubert AE, Beckers F, Verheyden B. Cardiovascular function and basics of physiology in microgravity. Acta Cardiol 2005; 60:129–151. doi: 10.2143/AC.60.2.2005024 15887469 10.2143/AC.60.2.2005024

