# Correction: Heritability of the dimensions, compliance and distensibility of the human internal jugular vein wall

**DOI:** 10.1371/journal.pone.0197526

**Published:** 2018-05-11

**Authors:** Adam Domonkos Tarnoki, Andrea Agnes Molnar, David Laszlo Tarnoki, Levente Littvay, Emanuela Medda, Corrado Fagnani, Antonio Arnofi, Filippo Farina, Claudio Baracchini, Giorgio Meneghetti, Giacomo Pucci, Giuseppe Schillaci, Maria Antonietta Stazi, György L. Nadasy

The seventh author’s name is spelled incorrectly. The correct name is: Antonio Arnofi.

Affiliation 4 for authors Emanuela Medda, Corrado Fagnani, Antonio Arnofi, and Maria Antonietta Stazi is incorrect. Affiliation 4 should be: Centre for Behavioural Sciences and Mental Health, Istituto Superiore di Sanità, Rome, Italy.
