# Supplementary material for: Heritability of the dimensions, compliance and distensibility of the human internal jugular vein wall
Source: PLoS One. 2018 Mar 21;13(3):e0192948. doi: 10.1371/journal.pone.0192948 (PMC5862397; doi:10.1371/journal.pone.0192948)
Supplement: S1 Table — Original data measured on twins. Anonimous Italian twin registry codes are shown. F, female, M, male, MZ, monozygotic, DZ, dizygotic twin pairs. Sit, sitting, Sup, supine body positions. Vals, during 60 mmHg Valsalva test, no Vals, without it with normal breathing. MLD, mediolateral diameter, APO anteroposterior diameter, in mm. (PDF) [file pone.0192948.s001.pdf]

ID	M/F	MZ(1) DZ(2)	yr of birth	Inner jugular vein Left Sit no Vals MLD	Left Sit no Vals APD	Right Sit no Vals MLD	Right Sit no Vals APD	Left Sit Vals MLD	Left Sit Vals APD	Right Sit Vals MLD	Right Sit Vals APD	Left Sup no Vals MLD	Left Sup no Vals APD	Right Sup no Vals MLD	Right Sup no Vals APD	Left Sup Vals MLD	Left Sup Vals APD	Right Sup Vals MLD	Right Sup Vals APD
353613A	F	1	1983	1,9	4,4	2,5	1,1	11,5	11,3	19	24,7	9,2	10,4	10,8	16,7	9,9	8,6	20,3	26
353613B	F	1	1983	2,2	5,2	4	7,8	13,9	16,4	12,9	17,1	9,5	12,6	10,3	14	15	17,2	15,2	23,3
376696B	F	2	1983	3	8,3	2,3	4,1	13,4	13,4	8,1	8,7	10,2	13,4	6	10,1	14,6	16,3	9,8	10,9
376696A	F	2	1983	6,2	2,8	6,2	2,2	11,9	13,4	12,7	16,1	8	10,8	11,3	7,7	12,7	15,7	13,6	21,7
825792B	F	2	1983	5,9	3,3	3,1	2,2	8,9	9,8	12,6	17,1	7,5	10,2	5,5	10,1	8,9	8,5	14,2	18,1
825792A	F	2	1983	4,2	1,9	4,2	2,1	16,7	18,1	11,1	13,1	15,1	17,4	10,8	17,1	16,6	15,8	12,1	21,3
574587A (102A)	F	1	1936	6,1	6,5	11,5	13	10,3	10,4	14,7	15,6	9	9,4	6,1	11,9	10,2	10,6	13,6	22,1
574587B (102B)	F	1	1936	6,6	10,3	11,6	15	11,1	12,1	14,1	32,6	9,5	14	8,5	13,4	16,8	11,1	14,9	35,2
705018A (107A)	M	2	1969	7,2	3,9	1,5	5,2	8,5	12,2	15,6	15,4	9,4	6,2	4,1	8,7	26,7	12,6	18	13,7
705018B (107B)	F	2	1969	5,4	2,1	4,5	2,7	12,1	11,8	14	15,3	8,7	11,7	8,8	11,7	11,9	14,1	11,313	13
253599A (104A)	F	1	1945	8,2	9,6	13,5	14,5	13,9	14	10,3	22,7	12,8	13,1	10,9	19,6	13,7	13,7	14,2	22,9
253599B (104B)	F	1	1945	14,4	17,1	7,7	6,9	12,1	18,1	10,3	14,4	10,9	17,4	6,5	18	16,9	26,7	9,8	17,5
097900A	F	1	1973	2,2	4,7	2,1	5,5	12,5	16,5	11,5	17,4	8,2	11,6	11,8	15,4	14,1	17,9	19	29,4
097900B	F	1	1973	2,4	5,2	5,3	2,6	12,6	15	15,8	17,7	11,2	17,8	12,9	10,8	15,7	18,2	17,6	22,6
824611A (114A)	M	2	1973	1,3	6	2	8,3	11,6	9,8	13,8	16,7	7,9	5,4	7,2	16,5	10,4	12,9	15	18,5
824611B (114B)	M	2	1973	8,9	11	4,2	1,4	11,5	14,8	9,6	15	7,3	11,3	9,9	13,9	15,3	14,1	14,9	18,7
592784A (113A)	F	1	1960	2,8	8,2	1,9	5,6	12,6	15,8	18,6	20,9	7,8	13,4	13,9	12,8	13,2	16	16,5	22,6
592784B (113B)	F	1	1960	3,5	5,8	1,6	2,8	15,4	15,8	10,8	15,5	8,6	14,7	8,9	14,5	12,4	12,4	13,2	16,5
390430B	F	1	1985	1,8	3,5	1,2	1,6	10,8	11,9	7,7	9,6	9,7	11	7,4	11,8	11,5	12,5	8,9	10,2
390430A	F	1	1985	1,9	2,1	1,3	2	9	10,1	11,9	13	7,6	7,7	6,2	11,6	9,4	9,7	12	16,3
775289A	M	1	1970	1,4	3,6	1,4	4,1	12,2	17,2	12,5	13,5	5,7	9,7	9	13,4	10,4	17,4	12,7	21,9
775289B	M	1	1970	3,4	1,2	2,1	4,4	8,4	11,2	14,1	20,2	4,6	9,9	5,1	10,7	8,7	12,2	10,5	13,8
778802B	M	1	1975	1,1	1,8	2	4,3	9,5	11,3	12,5	15,7	8,6	10,5	8,7	17,1	10	15,4	13,5	22,3
778802A	M	1	1975	1,8	5,1	0,9	2,8	7,9	13,5	10,7	12,6	10,1	13	8,2	21,1	13,6	16,1	12,8	15,8
049622A (117A)	M	1	1959	4,9	11,1	10,1	10,7	13,2	11,9	11,3	12,3	8,8	14,2	10,2	13,9	12,2	14,3	12,3	18,7
049622B (117B)	M	1	1959	2	2,9	2,1	3,7	12,5	12,1	16,1	21,4	8,4	13,9	5,8	16,5	11,6	15,2	18,1	20,8
723282A (108A)	M	2	1942	3,3	8,2	2,4	2,4	11,2	17,9	5,8	11,4	8,9	15,6	4	17,4	11,8	23,5	17,9	31
723282B (108B)	F	2	1942	4,7	2,4	2,6	4	14,1	23,2	14	15,7	9,8	14,6	9,5	19,3	13,2	19,4	10,8	15,3
785614B	M	1	1970	1,9	3,1	2,7	5,1	8,8	8,6	13,5	13,8	9,9	6,7	10	17,9	8,7	10,9	17,2	22,6
785614A	M	1	1970	5,6	6,7	3,5	6	8,1	9	14,3	17,9	4,2	9,1	6,4	15,8	7,2	12,2	12,4	20,2
328461A (118A)	F	1	1973	2,9	10,8	1,6	2,6	9,2	11,7	11,7	17,1	5,2	14	6,9	12	11,5	14,9	10,6	16,5
328461B (118B)	F	1	1973	2,3	4,3	2,2	6,4	12,2	15,8	9,5	15	3,5	8,6	2,5	11	8,4	15,8	10,2	15,6

543724A (121A)	F	1	1947	3	7	2,4	6,8	12,7	14,4	10,4	13,3	7,1	15,1	11,2	8	13,2	4,3	11,6	15,4
543724B (121B)	F	1	1947	3,6	6,8	3,8	9,3	6,5	9	14,8	17,3	5,4	8	4,8	11,8	8,2	9,1	17,5	24,3
730734A (128A)	F	2	1982	1,5	3,8	2,4	3,1	10,1	10,8	9,8	10,9	8,7	10,8	7,2	12,2	9,7	10,3	9,6	11,8
730734B (128B)	F	2	1982	1,8	5,5	1,7	3,7	10,7	10,9	9,4	11,6	8,3	10,2	8,9	15,7	10,2	11,8	9	12,7
485143A (109A)	F	2	1963	6,9	10,9	3,4	5,4	9,9	10,2	15,5	17	11,1	12,8	14	18,2	11,1	11,1	17,3	22,8
485143B (109B)	F	2	1963	6,5	7,9	4,1	0,3	10,9	9,8	17	16,2	6,1	8,5	12,1	17,1	12,5	14,8	14	16,4
820857A (103A)	M	2	1955	6,3	13	4,7	11,4	14,5	15,1	18,5	21,5	9,5	13,6	8	17	14,9	16,9	16,5	27,2
820857B (103B)	F	2	1955	11,8	13,5	2,5	5,4	11,4	15,1	10	19,7	3,8	10	6,3	10,2	14	18,6	16,5	20,6
335917A (119A)	M	2	1970	4,8	5,1	4	6,1	13,5	17,5	10,2	14,5	9	13,5	7,2	20,3	8,9	18	10,4	21,9
335917B (119B)	M	2	1970	2,2	4,5	3,3	5	14,8	16,4	7,6	14,1	8,1	14,6	6,7	11,4	15,8	14,1	11	18,3
532611A (111A)	F	2	1971	5,9	10,8	1,9	4,7	10	12,8	11,5	13,4	5,3	8,9	6,5	10,6	8,8	10	9,3	14
532611B (111B)	F	2	1971	2,1	6	1,4	5,2	15,3	20,1	6,8	12,4	10,6	16,8	7,4	10,7	17,5	21,2	10,4	18
078023A	F	1	1983	1,1	3,4	2,2	5,4	9,6	12	8,1	11,2	10,3	10,9	8,2	13,8	9,7	12,5	9,5	11
078023B	F	1	1983	3,2	5,7	1	3,5	9,3	10,6	7,8	10,6	8,1	10,3	10,8	13,1	10	10,8	8,4	13,1
339677A (126A)	F	1	1986	5,3	1,4	4,9	2,9	13,1	14,4	10,8	14,9	10,8	10,4	11,7	18	14,6	15	12,4	17,8
339677B (126B)	F	1	1986	1,2	3,7	1,1	6,3	8,6	7,6	14,7	15,4	9,4	7,7	10,8	18,9	9,5	10,6	15,1	26,3
809690B	M	2	1983	2,2	5,1	2,3	7,2	11,9	15,7	12,5	22,4	8,3	15,1	8,4	16,1	112	13,6	12,9	22
809690A	M	2	1983	3,5	6,4	2,9	4,9	9,8	12,2	12,5	15,3	7,7	10,5	8,4	11,6	11,8	12,5	10,4	23,1
117354A (105A)	F	1	1950	9,7	12,8	9,4	13,9	13,4	13,2	16,6	19,5	9	13,1	12,3	19,3	12	12	14,2	20,2
117354B (105B)	F	1	1950	2,3	5,6	4	7,6	13,1	15,7	22	20	7,3	13,1	13,1	20,7	14,1	18,1	18,9	26,4
063001A (122A)	F	2	1963	8,1	6,3	4,6	6	11,9	13,2	14,8	20	7,3	11,3	11,3	17,7	11,6	14,4	15,2	31,2
063001B (122B)	F	2	1963	2,6	3,7	2,3	5,1	8,7	8,3	12,9	18,7	6,8	9,4	12,9	20	7,9	8,8	15,8	22,4
224012A (127A)	F	1	1980	3,3	9,5	0,9	5,7	12,9	12,5	6	8,2	6,9	11,9	5,2	9,8	8,7	15,1	6	10,5
224012B (127B)	F	1	1980	6	8,8	8,3	10,4	7,3	7,5	12,1	15,7	5,8	8,7	6,6	11,5	7,7	8,3	10,3	17,3
059299B	M	1	1975	1,5	3,4	3,5	5,7	12,5	11	13,1	20,6	7	8,9	8,6	17,2	10,1	15,6	16	24
059299A	M	1	1975	2,3	8,1	1,7	5,5	14,1	16,9	13,7	13,9	4,6	12,6	4,2	14,1	13,4	17,7	12,4	16,9
273357B	F	2	1983	0,7	5,2	1,6	5,9	11,7	15,6	12,2	18,4	4,2	7,2	4,6	14,9	6,3	7,9	14,5	23,3
273357A	F	2	1983	4,1	6,8	2,7	7,9	14,6	14,9	13,3	20,2	8,5	14,8	8,6	16,7	14,4	14,4	10,9	28,7
328873A (124A)	M	1	1958	2,7	6,4	3,1	6,3	10,5	10,9	17,9	18,6	10,4	16,2	12	17,1	13,6	15,2	18,2	26,7
328873B (124B)	M	1	1958	5,2	1,7	3,8	2,5	9,7	16,5	13	19,7	111,8	15,5	10,3	26,4	11,4	19,4	11,2	41,4
649070A (120A)	M	1	1970	1,9	7,7	2,5	5,8	8,4	6,6	9,2	9,7	5,1	11,5	6,3	15,3	7,1	10,8	15,7	19,4
649070B (120B)	M	1	1970	8,1	11,6	1,9	3,5	11,8	16,1	12,9	15,8	8,3	13,8	11,8	19,3	11	14,3	14	18,7
1A	F	2	1964	4,68	5,27	3,11	4,55	14,33	13,99	12,36	14,7	10,89	15,11	11,74	13,87	12,57	23,08	12,45	17,64
1B	F	2	1964	9,15	6,76	3,23	5,91	8,83	12,74	18,33	22,2	7,2	10,47	6,23	12,66	14,89	18,85	13,9	28,53
2A	F	2	1942	4,97	3,12	3,15	5,69	6,36	9,53	18,82	21,68	10,98	17,1	13,95	22,05	13,33	19,9	18,91	20,84
2B	M	2	1942	5,74	4,39	5,52	4,72	22,34	11,88	21,25	22,99	7,6	8,55	7,6	16,88	10,05	14,02	17,48	25,29
6A	M	2	1974	2,56	3,43	6,98	3,51	11,14	14,48	12,79	23,67	9,41	10,68	12,15	18,9	13,79	12,97	16,51	25,45
6B	M	2	1974	2,14	3,99	3,32	8,94	10,14	28,12	12,9	10,82	4,81	8,09	11,14	6,59	8,31	8,97	13,4	13,53
9A	F	1	1971	2,95	4,62	2,53	3,53	13,07	15,74	15,24	18,19	7,94	10,14	10,4	12,62	12,57	13,7	15,48	24,9

9B	F	1	1971	1,55	4,21	5,58	5,9	11,73	12,08	15,32	16,72	8,46	9,77	11,83	19,82	13,43	16,31	15,1	18,75
10A	F	2	1987	3,53	8,51	1,53	6,22	12,82	13,05	12,38	22,32	10,97	27,93	14,2	20,97	14,47	16,93	16,32	24,72
10B	M	2	1987	2,4	5,53	2,92	8,37	8,59	12,37	14,4	18,15	7,02	11,65	8,35	15,52	12,43	14,12	17,01	21,5
051666A (11A)	F	2	1949	8,03	8,88	2	2,1	10,42	11,72	14,5	13,7	6,12	9,46	8,92	9,35	10,5	13,43	13,8	15,9
051666B (11B)	F	2	1949	7,6	8,68	2,66	3,66	105,1	11,48	8,96	9,78	8,59	10,32	5,77	10,43	9,15	15,38	7,97	8,33
12A	F	1	1945	3,1	4,78	3,02	6,07	14,77	25,77	18,26	19,74	14,71	21,93	12,17	15,25	23,54	29,09	21,05	20,42
12B	M	1	1945	4,85	3,06	1,97	3,53	15,48	15,53	23,29	28,63	11,09	17,53	9,55	20,48	16,39	19,17	22,06	32,58
13A	M	1	1959	1,79	6,57	1,71	5,21	13,2	19,69	18,7	29,14	13,79	16,27	13,89	19,75	15,44	22,41	19,1	26,13
13B	M	1	1959	1,21	7,07	0,79	9,66	8,47	19,5	14,45	21,99	6,12	14,35	10,11	14,26	12,88	20,7	15,45	24,74
14A	F	2	1964	6,49	8,2	6,4	4,75	16,75	17,05	15,9	24,8	11,89	16,35	15,27	25,92	16,51	16,51	17,36	23,66
14B	F	2	1964	6,6	11,64	5,74	8,14	15,27	16,72	19,38	29,72	12,8	17,63	14,35	16,79	14,63	19,79	22,97	29,3
15A	M	2	1965	4,26	6,76	5,01	5,85	4	6,91	8,57	9,94	8,15	10,11	7,82	18,18	8,22	9,28	16,39	13,96
15B	M	2	1965	3,97	2,77	2,9	6,17	5,24	3,38	5,06	7,04	9,52	17,06	7,05	16,58	15,26	14,17	10,6	16,26
16A	F	2	1962	7,29	10,13	3,2	6,6	13,06	14,5	10,7	17,72	8,51	11,39	9,71	20,2	12,99	20,42	17,65	28,59
16B	M	2	1962	8,76	8,62	1,41	3,03	15,6	15,09	10,9	17,2	12,7	14,6	10,51	12,52	15,54	23,58	18,03	19,84
17A	F	2	1941	2,1	3,53	2,84	3,75	15,01	18,87	14,53	13,64	15,35	14,99	12,59	17,68	15,12	16,99	13,64	17,23
17B	M	2	1941	2,51	5,42	5,25	7,03	10,06	11,06	17,92	21,66	11,99	21,92	6,94	17,48	10,93	15,53	19	23,11
19A	M	2	1937	1,88	7,68			13,39	10,97	17,63	21,21	13,39	10,97	16,22	28,95	19,23	18,73	18,55	20,47
19B	M	2	1937	6,4	12,52	8,13	4,94	14,28	18,06	22,63	29,69	15,83	24,49	17,35	33,47	19,55	25,76	20,83	38,73
20A	F	1	1972	3,04	6,07	2,56	5,72	13,69	13,65	15,76	17,56	11,51	12,77	11,29	13,2	13,34	15,18	15,03	18,87
20B	F	1	1972	3,49	5,23	4,85	7,47	11,06	12,11	15,17	19,78	10,8	13,28	10,19	19,95	9,84	13,76	13,77	23,57
21A	M	1	1969	6,56	3,44	3,4	6,75	14,49	19,52	19,08	24,84	11,71	16,74	9,23	17,83	18,82	20,65	12,99	21,77
21B	M	1	1969	2,75	5,69	2,95	7,32	11,24	13	19,2	26,76	9,06	13,75	8,75	18,78	13,92	17,31	18,98	25,07
23A	F	2	1945	8,01	5,69	0,92	5,13	18,12	17,56	7,37	11,52	11,45	13,04	6,09	10,26	10,84	16,79	8,18	9,51
23B	M	2	1945	5,87	12,93	4,32	3,19	22,54	25,77	15,2	17,74	18,57	33,61	13,58	19,26	24,45	31,56	15,62	20,41
24A	F	1	1945	9,41	10,4	7,24	7,83	10,42	13,43	12,01	20,3	10,27	15,87	10,97	18,75	11,59	14,89	18,33	25,98
24B	F	1	1945	7,31	6,67	4,21	3,96	16,25	19,26	22,06	32,9	12,5	19,46	14,48	23,6	16,95	31,62	20,14	35,11
26A	F	2	1970	2,2	2,33	3,37	11,5	8,36	8,61	15,59	16,01	6,33	8,79	12,36	20,11	7,19	8,71	16,01	24,1
26B	F	2	1970	2,97	5,95	1,69	4,69	13,65	15,1	10,49	10,43	6	12,65	6,34	10,05	12,01	16,02	10,79	13,68
28A	F	2	1986	1,89	4,36	3,72	8,07	16,4	19,83	13,49	15,64	8,36	12,66	9,77	11,85	13,49	13,73	14,72	19,95
28B	M	2	1986	3,97	9,17	2,47	5,34	13,98	15,46	15,14	21,71	5,79	13,06	9,01	13,33	15,16	20,09	13,84	17,21
29A	F	2	1949	3,06	1,69	4,26	6,19	12,11	11,95	16,1	19,31	7,84	11,49	11,08	18,65	12,39	13,34	12,56	19,37
29B	M	2	1949	5,7	3,45	6,28	2,67	11,95	17,19	15,98	16,87	12,24	15,51	9,2	17,29	12,73	23,48	12,67	18,41
31A	F	2	1953	2,68	6,22	3,05	4,64	11,39	14,33	17,98	14,59	6,07	13,04	13,05	25,84	12,08	15,8	17,7	27,53
31B	M	2	1953	2,69	3,92	2,33	7,47	13,14	13,36	19,04	21,07	12,08	14,27	13,29	21,81	12,24	15	17,97	17,92
34A	M	2	1961	4,67	3,68	3,07	5,06	17,77	26,92	21,44	25,92	16,45	22,92	16,88	26,99	17,49	24,19	20,72	28,51
34B	F	2	1961	6,55	9,42	5,86	4,71	12,26	14,31	12,79	12,71	10,48	12,98	8,02	12,56	10,65	15,24	10,75	15,33
38A	F	1	1959	2,27	4,05	2,25	4,39	12,52	14,87	13,7	15,39	9,93	12,15	11,59	17,29	12,64	15,2	13,13	21,61
38B	F	1	1959	2,14	5,38	4,43	6,53	6,05	8,54	19,39	21,52	6,37	9,08	15,75	19,35	9,71	11,11	19,64	25,4

40A	M	1	1977	4,34	8,6	2,1	11,6	14,22	22,97	15,28	23,26	10,3	19,59	10,66	24,09	19,55	29,14	22,66	34,64
40B	M	1	1977	3,09	6,14	3,16	9,22	15,56	25,24	17,55	27,65	9,84	14,64	13,21	21,75	17,72	23,46	19,92	26,41
43A	F	1	1959	8,15	2,53	1,79	6,96	18,57	23,79	13,34	13,98	7,5	11,89	4,72	10,05	16,99	28,9	11,17	17,65
43B	F	1	1959	3,47	5,23	2,14	3,44	14,41	22,74	14,89	18,75	7,02	15,91	8,04	18,75	14,5	20,93	16,43	18,62
44A	F	2	1970	8,13	12,34	2,01	3,94	15,26	16,79	15,12	17,45	8,23	11,76	9,77	15,62	14,35	17,04	13,2	17,24
44B	F	2	1970	1,83	1,48	3,48	6,78	14,92	13,69	5,77	11,22	7,73	11,24	9,13	14,96	12,78	18,92	11,18	21,11
45A	M	1	1975	3,39	4,2	2,18	4,71	18,47	15,75	16,93	17,95	6,69	11,83	6,12	14,19	17,37	17	20,07	29,71
45B	M	1	1975	6,28	15,26	1,88	5,88	17,54	23,78	22,39	33,37	5,52	9,62	11,07	21,29	13,25	20,14	21,52	36,48
45C	M	1	1975	3,47	7,8	3,59	9,79	19,89	23,52	21,74	26,13	3,68	9,4	3,73	8,42	14,18	24,56	14,58	28,27
264306A (1A)	M	1	1990	2,3	3,2	2,6	11,5	13,6	17,3	22,1	24,3	11,2	12,2	20,6	24,1	14,5	15,5	22,3	28,4
264306B (1B)	M	1	1990	7,5	10	3,5	7,7	13,3	14	15,4	21,4	10,2	14,9	13,3	20,7	14,8	13	14,8	21,5
497982A (2A)	F	2	1966	2,6	3,5	1,6	5,9	14,5	29,3	9,6	21,7	11,5	20	7,8	12,1	16,9	25,4	15,3	27,7
497982B (2B)	F	2	1966	10,6	10,7	3,1	6,8	16,3	18,9	17,6	23,3	9,6	12,9	9,7	14,8	15,5	20	13,4	25,3
506754A (3A)	M	1	1987	1,6	8,3	1,2	4,4	17,1	20	13,6	18,2	8	15,8	7	13,6	17	20,6	12	18,8
506754B (3B)	M	1	1987	2,5	2,2	1	5,7	15,4	20	14,5	18,7	6,5	9,9	3,7	14,8	13,7	20,5	14,8	26,9
783104A (4A)	F	2	1980	1,8	3,6	2,6	6,4	12,4	12,9	21,6	22,3	5,1	8,8	8,4	15,8	12,2	14	19,1	31,8
783104B (4B)	F	2	1980	2,2	7,2	3,2	9,2	9,3	10,7	15,7	19	6,1	10,1	10	18,3	10,2	11,3	15,6	23,1
028227A (5A)	M	2	1992	3,6	6,3	1,4	3	16,2	17,8	16,9	21,3	8,2	15,8	2,2	9,8	16	19,3	15,6	19
028227B (5B)	M	2	1992	3,7	7,6	1,6	5,4	17,4	23,8	15,2	16,3	3,5	9,7	2,9	13,3	16,6	23,4	13,1	19,9
024927A (6A)	M	1	1984	3,2	6,7	3,3	6,9	12	15,6	13,3	13,9	13,2	14	10	14,1	13,5	13,4	13,5	15,6
024927B (6B)	M	1	1984	2,5	4,3	5,2	6,1	9,4	8,4	20,6	20,9	7,3	9,8	9,7	16	7,8	9,7	15,2	20,6
547050A (7A)	M	2	1975	2,4	3,5	2	5,2	17,7	17,6	14,5	15,9	9,3	15,3	6,7	13,9	17,2	18,5	15	15,8
547050B (7B)	M	2	1975	1,5	6,5	4,6	4,6	14,8	19,6	14,9	16,8	7,5	10,8	7,3	10	11	15,3	14,5	18,7
759990A (8A)	F	1	1971	6,6	8,5	6	5,9	9,5	10,5	9,2	9,7	8,9	10	7,9	9	12	12	11,4	16
759990B (8B)	F	1	1971	7,9	6,4	1,3	3,7	15,2	15,9	8,8	10,3	11,6	13,4	6,5	9,2	12	13	8,7	11,3
380167A (9A)	F	2	1965	5	2,7	5,2	11	10,4	11,2	18	22,9	8,8	11,8	10,9	18,8	10,5	11,8	17,9	23,3
380167B (9B)	F	2	1965	2,1	6,1	0,93	3,4	12,8	12,3	8,5	10,1	8,3	14	5,9	13,4	11,7	15,9	10	16,8
375614A (10A)	F	2	1977	5	11,8	2,3	4,1	14,5	14	17,9	19,8	8,5	13,4	8	17,8	13,4	14,3	14,6	26,2
375614B (10B)	F	2	1977	3,8	8,2	2,6	6,5	9,5	10,2	11,7	12,4	5,6	10,2	5,2	10,4	9,9	9,9	11,8	18,5
134956A (11A)	M	1	1972	3,2	5,2	4,3	3,9	17,1	21,6	24	22,8	11,8	23,8	13,6	18,8	15,2	26,1	16,4	27,8
134956B (11B)	M	1	1972	5,5	10,3	4,1	5,6	12,5	13,7	14	13,2	12,7	23,5	11,6	13,8	24,8	31,1	14,4	14,8
975788A (12A)	F	2	1994	1,2	5,4	2,5	5,5	9,4	11,1	10	11,7	4,6	11,1	3,2	11,2	8,5	12,1	7,7	15,1
975788B (12B)	F	2	1994	3,3	5,4	4,4	5,8	8,3	11,8	11,2	11,8	7,9	10,2	9,3	14,9	8,7	9,6	12,2	20,2
698396A (13A)	F	2	1978	2,2	5,3	2	4,2	15	11,8	14,1	19,8	11,6	16,2	13,8	30,7	13,3	16,4	16,4	28,8
698396B (13B)	F	2	1978	2,3	6,4	2,4	7,7	15,3	14,4	20,8	23,2	7,8	10,6	13,4	15	12,1	11,6	20	29,6
772160A (14A)	F	1	1977	2,4	6,5	1,1	6,5	8,8	8,3	8,9	11,8	6,5	9,5	6	11,1	8,3	9,2	9,1	11,9
772160B (14B)	F	1	1977	1,9	4,5	1,5	4,6	11,6	13	7,3	9,7	9,6	11,4	5,5	8,5	9,2	11,6	7,9	8,3
545496A (15A)	F	1	1967	1,7	6,3	1,8	4,3	10,1	9,3	9,7	14,1	5	8	10,8	15,1	8,1	9,8	16,4	21,8

545496B (15B)	F	1	1967	4,4	8,5	2,1	3,9	10,2	10,1	11,6	13,7	6,8	12,4	4	11,1	8,4	10,5	10,4	16,3
127884A (16A)	M	2	1965	4,9	6,9	5	4	16,9	16	13,4	16,3	8,6	14,1	8,5	16	17	17,8	13,3	15,5
127884B (16B)	M	2	1965	3,1	4,2	1,5	4,2	13,6	14,6	10,2	12,5	10,9	14,1	5,9	13,5	16,2	16,8	9,9	11,2
648182A (17A)	F	2	1985	2	9,3	2	5,9	10,7	12,9	10,7	16,1	4,1	8,4	5,2	10,2	11,2	13,9	13,1	20,9
648182B (17B)	F	2	1985	3	4,4	2,3	4,4	16,4	21,9	15,1	18,5	11,2	17,7	9,5	11,2	13,7	19,5	12,9	20,9
611831A (18A)	M	1	1968	3,7	7,2	3,3	6,1	18,4	16	10	11,9	12,8	18,1	12,1	16	13,2	18	13,3	17
611831B (18B)	M	1	1968	4,7	8	2,5	6,7	10,6	12,6	18,3	19,4	9,5	11	10,9	16	9,8	10,8	14,3	24,4
176734A (19A)	F	1	1979	2,4	3,7	1,6	8,5	12	15,7	14,8	18,4	8,9	13,5	9,3	16,4	12,1	14,7	13	21,8
176734B (19B)	F	1	1979	3,2	7,4	2,5	6	12	14,5	15,5	24,5	6,9	13,1	11,1	19,6	12,1	17,2	17,8	26
546139A (20A)	F	1	1972	0,82	1,7	1,8	13,3	4,5	6,4	6,5	15,9	6,2	6,7	6,4	17,5	3,8	7,9	9,8	16,4
546139B (20B)	F	1	1972	5,1	5,5	3,6	5,1	6,9	6,7	10,2	18	7,1	7,1	11	16,2	7,3	5,6	13,2	18,6
428455A (21A)	M		1982	2,5	4,7	1,7	5,2	16,1	22,5	18,2	24,6	9,2	16,2	5,3	15,1	17,3	20,5	18,1	26,9
428455B (21B)	M		1982	2,2	6,7	1,3	8,1	13,1	13,4	24	28	10,2	13,7	9,5	15,6	16,8	14,8	21,1	27,1
167150A (22A)	F	2	1979	2,8	8,8	2,8	6,7	12,9	15,6	16,6	18,2	8,9	13,1	9,7	18,6	13,2	14,6	15,5	18,9
167150B (22B)	F	2	1979	0,8	3,2	1,2	9,8	9,2	8,9	14,6	16,6	7	12	7,7	14	8,6	8,4	14,3	17,5
770385A (23A)	M	1	1979	3,6	5,7	4,6	6	14,7	12,8	15,8	13,5	9,4	13,3	10,1	13,8	12,2	10,8	14,2	18,7
770385B (23B)	M	1	1979	3,2	8,6	2,7	7	10,6	16,8	7,7	13,2	10,9	12,6	8,2	12,7	14	14,6	11,6	15,5
975758A (24A)	F	2	1970	13,2	16,8	4,1	10,3	14,9	16,9	15,6	17,6	6,5	15,4	4,3	15,2	16	18,5	13,5	22,7
975758B (24B)	F	2	1970	6,2	10,6	1,3	5,1	16,2	16,9	7,8	12,6	6,5	12,3	0,56	2,9	16,4	21,1	8,1	10,9
975758A (25A)	M		1971	8,9	12,9	3,3	5,7	21,3	24,1	16,7	22,9	14,1	19,3	11,7	20,3	19,7	23,2	19,3	25,4
975758B (25B)	M		1971	1,6	5,7	3,4	9,6	12,9	15,7	21,9	23,2	8,8	11,9	11,6	18,6	11,2	14,8	17,4	24,9

Supplementary Table I. Diameters of the inner jugular vein measured ultrasonographically.
Original data measured on twins. Anonimous Italian twin registry codes are shown.
F, female, M, male; MZ, Monozygotic, DZ, Dizygotic twin pairs.
Sit, sitting, Sup, supine body positions.Vals, during 60 mmHg Valsalva test, no Vals, without it with normal breathing.
MLD, mediolateral diameter, APD, anteroposterior diameter, in mm.
